# Gender Inequitable Masculinity and Sexual Entitlement in Rape Perpetration South Africa: Findings of a Cross-Sectional Study

**DOI:** 10.1371/journal.pone.0029590

**Published:** 2011-12-28

**Authors:** Rachel Jewkes, Yandisa Sikweyiya, Robert Morrell, Kristin Dunkle

**Affiliations:** 1 Gender & Health Research Unit, Medical Research Council and School of Public Health, University of the Witwatersrand, Pretoria, South Africa; 2 Research Office, University of Cape Town, Cape Town, South Africa; 3 Rollins School of Public Health, Emory University, Atlanta, Georgia, United States of America; Central Institute of Educational Technology, Canada

## Abstract

**Objective:**

To describe the prevalence and patterns of rape perpetration in a randomly selected sample of men from the general adult population, to explore factors associated with rape and to describe how men explained their acts of rape.

**Design:**

Cross-sectional household study with a two- stage randomly selected sample of men.

**Methods:**

1737 South African men aged 18–49 completed a questionnaire administered using an Audio-enhanced Personal Digital Assistant. Multivariable logistic regression models were built to identify factors associated with rape perpetration.

**Results:**

In all 27.6% (466/1686) of men had raped a woman, whether an intimate partner, stranger or acquaintance, and whether perpetrated alone or with accomplices, and 4.7% had raped in the last 12 months. First rapes for 75% were perpetrated before age 20, and 53.9% (251) of those raping, did so on multiple occasions. The logistic regression model showed that having raped was associated with greater adversity in childhood, having been raped by a man and higher maternal education. It was associated with less equitable views on gender relations, having had more partners, and many more gender inequitable practices including transactional sex and physical partner violence. Also drug use, gang membership and a higher score on the dimensions of psychopathic personality, namely blame externalisation and Machiavellian egocentricity. Asked about why they did it, the most common motivations stemmed from ideas of sexual entitlement.

**Conclusions:**

Perpetration of rape is so prevalent that population-based measures of prevention are essential to complement criminal justice system responses. Our findings show the importance of measures to build gender equity and change dominant ideas of masculinity and gender relations as part of rape prevention. Reducing men's exposure to trauma in childhood is also critically important.

## Introduction

Rape violates the right to bodily integrity and has an enduring impact on the health and social lives of victims, who are most commonly women and girls [1], [2]. Research on rape has predominantly focused on women and girl victims with survey research showing that 6–59% of women globally report sexual violence from an intimate partner [3]. A review published by the World Health Organisation on factors associated with being a female victim identified these as being youth, poverty, physical disability, mental vulnerability, substance abuse, prior victimisation and coming from a dysfunctional home [4]. These markers of vulnerability provide very little information about who rapes, under what circumstances rape is perpetrated and what reasons men give for perpetration. For successful rape prevention, a focus on victims is less helpful than understanding the men who commit rape.

### Literature on rape perpetration

Studies on rape perpetration have primarily been conducted in North America among two participant groups: college students and men convicted of rape. Much of the college men's research has not had the statistical power to focus on completed acts of rape [5], [6]. Exceptions include two surveys of American naval recruits [7], [8] and two quantitative studies with non-random samples of South African men [9], [10]. Research with small random samples of men in the general population has been conducted in Canada (n = 195) and the United States (n = 163) [11], [12]. These lacked the power to explore perpetration in depth, but nonetheless Abbey et al [12] found 24.5% of men reporting an act that would meet legal definitions of rape or attempted rape.

Theoretical understandings of rape perpetration largely come from the research with college students and convicted rapists. Particularly influential here has been the Confluence Model [13], [14], [15], which is a multi-factorial model that has been tested using structural equation modelling on several datasets. It shows two pathways influencing sexual assault perpetration: hostile attitudes towards women and sexual promiscuity-impersonal sex. Each path independently predicts perpetration, but they also work synergistically, and with men scoring highly on both paths being more likely to be sexually coercive.

In the first pathway, Malamuth et al show that men who have raped have cynical, adversarial and hostile ideas of male-female and intimate relationships, including feelings of shame (especially about sex) and inadequacy, which may be masked by anger, and an exaggerated need to control women [13]. These men show a greater acceptance of interpersonal violence and adversarial sexual beliefs [13], [14], [16]. They perceive women as hostile to them, mistrust women's affective expression, and are more likely to interpret assertiveness in women's interactions with them as hostile [17], [18]. Malamuth argues that such hostile cognitions are more likely to develop in the context of delinquent youth peer relationships and men who are sexually violent are more likely to report these [13], [14]. In turn, such delinquency is associated with experience of trauma in childhood, particularly sexual abuse and witnessing IPV. Pressure from peers to have sex may also encourage some men to force sex [12], [19], [20], and to do so more often [12].

In the second pathway, Malamuth describes how men who emphasise heterosexual performance, particularly with sexual conquest, as a source of peer status and self-esteem, may use various means, including coercion to secure sexual partners [13]. He argues that experience of trauma in childhood reduces the ability of men to form loving and nurturing attachments, and this results in an orientation to impersonal sexual relationships rather than sex in the context of emotional bonding and other short term sex-seeking strategies [13].

Malamuth paid scant attention to alcohol, yet it plays a role in a high proportion of rapes [12], [16], [21], [22]. It is regarded as a situational factor [16] , but also reduces inhibitions[23] (like some drugs, notably cocaine [24]), clouds judgement and enables a greater focus on the short term benefits of forced sex [12], [25]. It may also act as a cultural ‘time out’ for anti-social behaviour [16].

Since its original development the model was improved by acknowledging the attenuating effect of personality dimensions of empathy and being orientated towards others [26]. Sexually violent men, especially multiple offenders, are more likely to lack empathy or have remorse for their victims and to blame their victims for the rape [12], [16], [26], [27]. Knight & Sims-Knight [27] have further emphasised the impact of psychopathic personality and the role of childhood trauma in the development of sexual aggressive behaviour, and argued that adding these to the model enables it to fit the data slightly better than the original Confluence Model.

Research from South Africa supports many aspects of Malamuth at al's findings, with an association described between rape perpetration and childhood trauma, gang membership, transactional sex and having large numbers of sexual partners [9], [10], [28]. But there are also dissimilarities. The Confluence Model fails to discuss the social context that influences exposure to trauma in childhood and likelihood of involvement with gangs. Further the model uses gender categories uncritically and thus it lacks a gender analysis. Many authors have pointed to the importance of gender hierarchy in rape [29], [30]. A further area of critique is in their assumption that men's exposure to childhood trauma and consequent reduced ability to form nurturing attachments to women result in them seeking impersonal sex. Psychoanalytic analyses more strongly support an argument that trauma in childhood, particularly disorganised attachment would have resulted in a tendency towards borderline personality, rendering men unable to form stable attachments to women partners, vulnerable to splitting (i.e. switching between idealizing or demonising others), mood disturbances, problems with self image and disturbances of sense of self [31]. This would result in an inability to sustain stable adult relationships, rather than an inability to desire them [32].

### Masculinity, poverty and sexual violence

Among men who rape, the relevance of poverty and other factors are complex. The way men understand themselves is affected by their life circumstances and there is a dialogic relationship between their subject positions and social circumstances which are reflected in their actions [33], [34]. Starting with men's childhood, the links between childhood trauma and disorganised caregiver child bonding and poverty are well described. The increased risk of child abuse and neglect in homes where mothers are very young, single, unsupported and impoverished has been described in many settings [35], [36]. Yet research from South Africa has pointed to some complexity. It has shown that in a large sample of mostly rural young men, rape perpetration was more common among those who came from less poor backgrounds, had earned money and had more educated mothers [10]. Thus in a context of poverty, unemployment and low levels of education, it was the relatively less poor and disadvantaged who were more likely to rape. They suggest that the driving factor was a sense of entitlement, stemming from this relative advantage, that played out in a perverse way in a context in which few or none would be able to attain objectively high levels of material ‘success’. This resonated with the work of others who have described how in conditions of poverty young men form gangs and rape in a quest for gendered power in a context where traditional routes of attainment, such through fulfilling provider roles, are unattainable [37]–[39].

Several authors have argued that the relationship between poverty and rape perpetration is mediated through ideas of masculinity and the quest for ‘success’ [37], [38], [40]–[43]. Feminist scholars have shown how particular understandings of masculinity legitimate unequal and often violent relationships with women [33], [44], [45]. Connell describes the existence of multiple, mutable masculine positions and identities, arranged hierarchically with respect to each other (some are viewed as legitimate whilst others are censored), and superior to women. She argues that in a given setting, one ideal of masculinity is hegemonic, i.e. achieves dominance through a complex process which includes the consent even of those dominated [44]. Legitimacy may pertain at multiple levels – indeed what may be seen as hegemonic at one level may be subordinated at another (e.g. sub-cultural versus national level) [46]. Research from South Africa, a context of marked gender inequality, has shown that hegemonic masculine ideals give centrality to heterosexual performance, toughness and strength [47], [48]. Here heterosexual performance is predicated on an ability to control of women, and physical and sexual violence may be used as strategies to achieve and assert this. Gang rape can be viewed as an accentuated display of collective heterosexual performance in which the woman rape victim is ultimately objectified [10]. Whilst rape, or transacting sex, are not necessary in demonstrations of heterosexual prowess, both may be enacted in its course of performance and as evidence of control over women [49].

With most of the theory on rape perpetration developed in the United States, and exclusively in research with non-random samples of men, there is a considerable need to explore these factors among men in the general population to deepen our understanding of rape. This has been possible in research in South Africa in a survey that sought to incorporate factors described in the published literature, but also expand understanding of connections between rape and gender inequity and violence towards women, negative dimensions of life in childhood and involvement in crime. The aim of this paper is to describe the prevalence and patterns of rape perpetration in a randomly selected sample of men from the general adult population, to explore factors associated with rape and to describe how men explained their acts of rape.

## Methods

The study was undertaken in three districts in the Eastern Cape and KwaZulu-Natal provinces of South Africa. These form a contiguous area, and include rural areas with communally-owned land under traditional leadership, as well as commercial farms, small towns, villages, and a city, inhabited by people of all South African racial groups, several ethnic groups (predominantly Xhosa and Zulu) and socio-economic backgrounds.

The sample used a two stage proportionate stratified design to identify a representative sample of men aged 18–49 years living in the three districts. Using the 2001 census as the primary sampling frame, 222 census enumeration areas (EAs) were selected as the primary sampling unit, stratified by district and with numbers proportionate to district population size. The sample was drawn by Statistics South Africa. Households in each EA were mapped and twenty were systematically selected. In each household one eligible man was randomly selected to take part in the interview. Men were eligible for the study if they were aged 18–49 years and had slept there the night before.

Of the 222 selected EAs, two (0.9%) had no homes, and in five (2.3%) we could not interview because permission from the local political gatekeepers was declined (1) or we could not access any eligible home after multiple visits at different times of day (4). In all the latter EAs, we established that many households were ineligible due to age or absence of a man. We completed interviews in 215 of 220 eligible EAs (97.7%). We sampled a total of 4473 visiting points. Of these, 1353 (37.1%) were found to contain no eligible man, 2298 (51.4%) contained at least 1 eligible man, and 822 (18.4%) could not be rostered for eligibility after a minimum of 3 attempts at contact. We completed interviews in 1737 of 2298 (75.6%) enumerated and eligible households.

Interviews were conducted in isiXhosa or isiZulu or English with data collected using self-completion on APDAs (Audio-enhanced Personal Digital Assistants), thus participants could hear and read each question and its response options. Interviews took 45–60 minutes to complete. Only one participant was unable to do this and he asked the fieldworker to enter his responses. During interviews the fieldworkers were in the room to help if needed. Interviews were conducted in private and family members were not around. The confidentiality was ultimately assured through self-completion.

### Measurement of rape

Rape perpetration was assessed using seven questions developed for the study and validated through cognitive interviewing, none of which actually used the word ‘rape’ [50]. They were modifications of those used previously in the Eastern Cape [10]. A typical item was “How many times have you slept with a woman or girl when she didn't consent to sex or after you forced her?” The questions additionally asked about having forced a (former) girlfriend or wife into sex, having forced a woman who was not a girlfriend or wife into sex, having sex with a woman who was too drunk to consent and having had sex with a woman together with other men when she didn't consent to sex or was forced or was too drunk to stop them. Men disclosing were asked the age they first raped and the age of their youngest victim. We also asked about attempted rape and participating in gang rape when they did not have sex. Men were then asked two questions about perpetration of rape of a man or boy. In this paper we consider a man to have ‘raped’ if he had indicated in any of the seven questions about rape of women that he had completed an act on one or more occasions.

Men who reported rape perpetration against a woman or man were asked a series of questions on antecedents and motivations and the ages of the victims and his age at the time. These were derived from qualitative research and pre-tested [50]. Each was presented as a statement and they were asked to respond on a 4-point Likert scale (from strongly agree to strongly disagree) indicating whether the statement was an explanation for why they did it. A typical item was “we wanted to have some fun”. These statements were asked separately for each type of rape or circumstance and there were some differences between the statements used for each type. Variables were derived to group these statements in motivation categories for those who strongly agree or agreed with a statement. A sexual entitlement variable included responses to statements about wanting to ‘have sex’, ‘I wanted her sexually’, ‘wanted to prove I could do it’ and ‘experimenting with sex’. An anger variable included responses to ‘I was angry with her’, ‘angry with someone else’ and wanted to ‘punish her’. A fun variable captured responses to ‘it was a joke or game’ or ‘we wanted to have some fun’… Statements also asked about whether he was ‘bored’, ‘forced or pressurised by friends’ (not asked for girlfriend/wife rape), had been drinking or ‘wanted to clean myself of sexual diseases’ (not asked for gang rape).

### Other variables

The questionnaire included categorical variables measuring age, education, race, employment, income and frequency of hunger due to lack of money. Questions on men's childhoods included items on whether and how often their parents worked and were at home and mother's level of schooling. Scales measured men's perceptions of the kindness of their mother (3-items, Cronbach's alpha = 0.75) and father (4-items, Cronbach's alpha = 0.87). A typical item was “I had a loving relationship with my mother/father while I was growing up”. A four point Lickert response scale was used (strongly agree, agree, disagree, strongly disagree).

Data on adverse experiences before the age of 18 were collected using a locally modified version of the short form of the Childhood Trauma Questionnaire [51], [52]. We assessed five dimensions of adversity: emotional neglect, emotional abuse, physical neglect/hardship, physical abuse and sexual abuse using a four point response scale (never, sometimes, often and very often) (Cronbach's alpha 0.79). A typical question was “before I reached 18 one or both of my parents were too drunk to take care of me”. Men were asked if they had been teased or harassed as a child, and whether they had ever been raped by a man (“persuaded or forced to have sex when you did not want to”).

Attitudes towards gender relations were measured using 10 items from the Gender Equitable Men scale [53] (Cronbach's alpha 0.78). A typical item is “There are times when a woman deserves to be beaten”. A high score denotes more equitable attitudes. Adversarial sexual beliefs were measured using a six item scale, a typical item is “When women work they are taking jobs away from men” [54] (Cronbach's alpha 0.80). A higher score indicates less adversarial attitudes. Hostility towards women was measured using a 5 item scale, a typical item being “When it really comes down to it a lot of women are deceitful” (Cronbach's alpha 0.77). A high score denotes more hostility. Rape myths were measured on a 4-item scale where a higher score denoted more myth belief. A typical item was “in some rape cases women actually want it to happen” (Cronbach's alpha was 0.76).

Practices of gender relations were measured through questions about number of sexual partners, and about transactional sex with women, defined as sex that was primarily motivated by a desire for material gain on the part of the woman. This was defined as providing food, cosmetics, clothes, transportation, items for children or family, school fees, somewhere to sleep, handyman work, or cash [55]. Men were asked about lifetime perpetration of physical intimate partner violence using the modified WHO violence against women instrument [3]. Specific acts of violence were asked about in five items ranging from slapping to threats with or use of a weapon.

Recent alcohol consumption in the past 12 months was assessed through a question on frequency of having 5 or more drinks per drinking day. Drug use was assessed through a question on how often the man had smoked dagga (cannabis) in the past 12 months. Men were asked if they had ever been in a gang.

Data were collected on two dimensions of psychopathy. Thirteen questions on Machiavellian Egocentricity and Blame Externalisation sub-scales of the Psychopathic Personality Inventory- Revised (PPI-I) were included. The Cronbach's alpha for the scales together were 0.83. A typical item on the Machiavellian Egocentricity sub-scale was “I get mad if I don't receive special favours I deserve” and on the Blame Externalisation sub-scale was “I have often been betrayed by people I trust”. Each has a 4 level response option (false, mostly false, mostly true, true). These were adapted and reproduced by special permission of the Publisher Psychological Assessment Resources, Inc., 16204 North Florida Avenue, Lutz, Florida 33549, from the Psychopathic Personality Inventory- Revised by Scott O. Lilienfield, Ph.D., Copyright 2005 by PAR, Inc. Further reproduction is prohibited without permission of PAR, Inc.

We asked four items to measure empathy, adapted from Abbey et al [12] (Cronbach's alpha 0.80). A typical item was “I am often touched by things that I see happen”. These had a five point response scale (doesn't describe me well – describes me well). Perceptions of life success were assessed with the following question: “If you compare your life circumstances overall now with those of the people you grew up with, would you say you have done much better for yourself, somewhat better, the same, less well, much less well?”

The school bullying score was an 8-item scale used to measure experiences with bullying at school with four level response options (never, sometimes, often and very often) (Cronbach's alpha 0.76). These questions were developed for the study. A typical item was “My school friends and I were a group and we would put pressure on a girl to date one of us until she agreed”.

We asked 11 items about lifetime experiences of participation in crime. These were modified for the local context from Tremblay et al [56] who developed them as a measure of delinquency in childhood. Eight of the items related to theft (Cronbach's alpha 0.81) and a typical item was “how often have you stolen an animal from someone?” The response options were never, once, 2–3 times and more often. Men were asked about weapons and arrests.

### Ethical issues

The men were informed about the study, given an information sheet and signed informed consent. As an incentive, they were given R25 (US $3.2) for the interview and those who gave a blood sample for HIV testing were given a further R25 (data not discussed in this paper). Consent for the interview, completion of the interview and the request for blood for HIV testing were performed in sequential stages so that a man who might decline to give blood for HIV could still agree to the interview. It was unlikely that being asked for blood was major deterrent to the interview as very few of those eligible who were asked for an interview declined, but many men did decline the blood sample. Since the questionnaire asked men to disclose a range of criminal acts and South African law does not privilege research data, interviews were conducted anonymously. No identifying details of the men or their households were kept after the interview and the consent forms could not be linked. Ethics approval was given by the Medical Research Council's Ethics Committee.

### Data analysis

The study design provided a self-weighted sample. Data files were collated and analyses were carried out using Stata 10.0. All procedures took into account the two stage structure of the dataset, with stratification by district and the EAs as clusters. The distribution of social and demographic characteristics, childhood experiences, experience of abuse and rape victimisation, attitudes, psychological measures, aspects of gender relations, substance use and engagement with other violent and anti-social behaviour variables by rape perpetration status were summarised as percentages (or means), with 95% confidence limits calculated using standard methods for estimating confidence intervals from complex multistage sample surveys (Taylor linearization). Pearson's chi was used to test associations between categorical variables. Motivations for raping were presented as percentages by type of rape. No efforts were made to replace missing data.

The age structure of the study sample was a little different from that of the two provinces based on mid-year estimates from Statistics South Africa [57]. Our sample was somewhat younger, with 51.5% of men aged 18–24 years compared with the expected figure of 36.1%. In order to show the impact of this difference on rape prevalence we present the prevalence (% and 95% confidence intervals) of disclosed perpetration of different types of rape among the study men as well as that calculated with the data weighted to the age structure of the population in the two provinces.

To account for clustering of men within EAs, we used random effects logistic regression models. We present two, one showing factors associated with having ever raped and the other factors associated with rape in the past year. All the variables shown in the preceding tables were candidates for inclusion in the models. Variables were entered in conceptual groups (as per the tables) and backwards elimination was performed with variables retained at a conservative p<0.2. The final models were then derived with variables retained at p≤0.05. We tested for interactions between retained variables and found none.

## Results

Half of the men interviewed (51.5%) were aged 18–24 years, 19.1% were aged 25–29 years, 11.7% aged 30–34 years and 17.6% aged 35–49 years. Just over a third of the men had completed school or attended a tertiary institution. Eighty-five percent were Black African, 9% were Indian, 2% White and 4% Coloured. Most men (61%) were single and two thirds either had no income or earned less than R500 per month.

### Prevalence and patterns of rape

In all 27.6% (466/1686) of men had raped a woman, whether an intimate partner, stranger or acquaintance, and whether perpetrated alone or with accomplices (weighted prevalence 27.9% 95% CI 25.3, 30.7). 21.4% (360/1681) had raped a woman who was not a partner (i.e. a stranger or acquaintance or family member) (weighted prevalence 21.7% 95%CI 19.4, 24.3). 14.3% (241/1681) had raped their current or an ex- wife or girlfriend (weighted prevalence 15.0% 95%CI 13.0, 17.3). 8.9% (149/ 1680) had been involved in a gang rape in which they had sex (weighted prevalence 8.8% 95%CI 7.4, 10.4). Seventy five men (4.7%) had raped a child under 15 years (weighted prevalence 4.2% 95%CI 3.4, 5.2). In addition, 16.8% (278) of men disclosed that they had attempted to rape a woman or girl but not completed the act (weighted prevalence 16.7% 95%CI 14.7, 19.0). The total proportion of men interviewed who had ever completed an act of rape or attempted rape was 33.0% (weighted prevalence 32.9% 95%CI 30.2, 35.8). The total proportion of men who had participated in a gang rape either having had sex or assisting the rape was 19.5% (weighted prevalence 18.6% 95% CI 16.5, 20.9).

**Figure 1:**
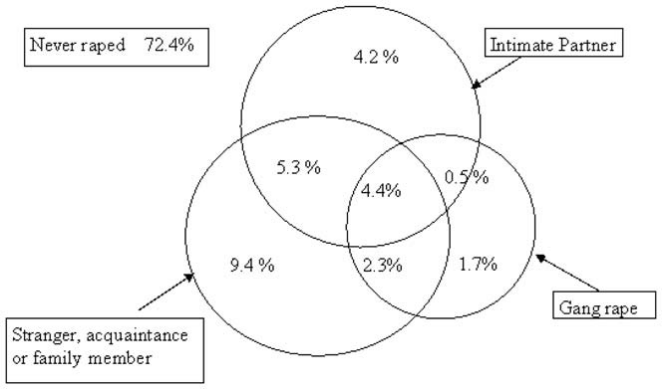
Venn diagram showing the overlaps between perpetration of rape of a girlfriend or wife, a non-partner and gang rape.

Figure 1 is a Venn diagram which shows the overlap between disclosure of perpetration of rape of an intimate partner, gang rape and rape of a stranger, acquaintance or family member. 14.9% of all men interviewed, and 53.9% (251) of men who had raped, had done so more than once. 4.7% (78) had raped in the 12 months before the interview. Asked the age when they (first) raped, 75% disclosed having done so before age 20.

### Factors associated with having raped

The social and demographic characteristics of men who had ever raped a woman, compared to those who had not are presented in table 1. Men who had raped did not differ by age or educational attainment, but there were significant differences in racial group, marital status, monthly income and in food insecurity. There were more Coloured men and fewer White men among those who had raped. More men who had raped were cohabiting than those who hadn't, and fewer were married. Fewer men who had raped were unemployed or had very low income, although a higher proportion reported that they sometimes or often went hungry due to lack of money.

**Table 1:** Distribution of the social and demographic characteristics, childhood experiences and rape victimisation by rape perpetration.

	Raped					Never raped					
	n	N	%	95% CI		n	N	%	95% CI		p value
age: 18–24 years	232	466	49.8	44.6	55.0	639	1220	52.4	49.0	55.8	0.180
25–34	161		34.5	30.2	38.9	365		29.9	26.9	32.9	
35–49	73		15.7	12.1	19.2	216		17.7	15.2	20.2	
Education: completed school	203	465	43.7	38.7	48.6	473	1212	39.0	35.6	42.5	0.104
Race: Black African	389	466	83.5	78.2	88.7	1047	1220	85.8	81.7	90.0	0.003
Coloured	32		6.9	3.8	10.0	38		3.1	1.5	4.7	
Indian	42		9.0	4.7	13.3	111		9.1	5.4	12.8	
White	3		0.6	0.0	1.4	24		2.0	1.1	2.8	
Marital status: married	90	462	19.5	16.0	23.0	277	1200	23.1	20.3	25.9	0.0001
cohabiting with woman	76		16.5	13.2	19.7	114		9.5	7.6	11.4	
divorced/widowed	24		5.2	3.2	7.2	36		3.0	2.0	4.0	
Single	272		58.9	54.5	63.2	773		64.4	61.1	67.7	
Monthly income: R0–500	271	456	59.4	54.6	64.2	824	1181	69.8	66.4	73.1	0.0003
>R 500	185		40.6	35.8	45.4	357		30.2	26.9	33.6	
Ever hungry due to lack of money	246	422	58.3	53.4	63.2	563	1140	49.4	45.9	52.9	0.003
**Childhood experiences**											
Perceptions of maternal kindness (mean score)	439		10.53	10.32	10.74	1133		10.91	10.81	11.01	<0.0001
Perceptions of paternal kindness (mean score)	440		11.18	10.86	11.50	1144		12.16	11.93	12.40	<0.0001
Mother never or rarely at home	210	461	45.6	40.4	50.7	454	1196	38.0	35.2	40.7	0.012
Father never or rarely at home	331	455	72.7	68.4	77.1	762	1168	65.2	62.4	68.1	0.006
Was teased and harassed as a child	314	465	67.5	63.1	71.9	599	1211	49.5	46.3	52.6	<0.0001
Childhood trauma scale (mean, high = more trauma)	443		21.09	20.52	21.65	1158		18.48	18.18	18.78	<0.0001
Mother's education: none	68	421	16.2	12.3	20.0	274	1078	25.4	22.5	28.3	<0.0001
some schooling	295		70.1	65.2	74.9	733		68.0	65.0	71.0	
completed school or higher	58		13.8	9.9	17.6	71		6.6	5.0	8.2	
Raped by a man	80	464	17.2	13.5	21.0	76	1204	6.3	4.9	7.7	<0.0001

The childhood experiences of men who had raped and those who had not are presented in table 1. There were notable differences, with men who had raped perceiving that their mothers and fathers were less kind, and had been completely or more often absent from home when they were growing up. A greater proportion had been teased or harassed at school or in the community as a child and they had a much higher mean score on a scale measuring childhood trauma (emotional abuse and neglect, physical abuse and neglect and sexual abuse). A further difference was that men who raped had more educated mothers, with a greater proportion having completed school or attended further education. The proportion of men who had themselves been raped by a man was nearly three times greater than that found among those who had not been raped (17.2% v. 6.3%).

There were significant differences in the gender attitudes held by men who raped compared to those who did not (table 2). Men who raped held less equitable views on gender relations and were more adversarial in their views about women. They more strongly adhered to rape myths and were more hostile towards women. In terms of gender practices men who had raped had many more sexual partners. The proportion with 20 or more partners in their lifetime was twice as large for men who had raped compared to those who had not done. They were more likely to have had transactional sex and had much more physical violence against a partner, with over half having been violent on multiple occasions.

**Table 2:** Distribution of gender attitudes, practices of gender relations, substance abuse and psychological measures by rape perpetration.

	Rape					Never raped					
	n	N	%	95% CI		n	N	%	95% CI		p value
**Attitudes**											
Gender equitable men scale (mean) (high = more equitable)	451		22.36	21.84	22.87	1165		23.81	23.43	24.19	<0.0001
Adversarial sexual beliefs score (mean) (high = less adversarial)	456		14.14	13.67	14.60	1180		14.79	14.48	15.11	0.018
Hostility towards women score (mean) (high = more hostile)	456		4.39	4.09	4.69	1178		3.96	3.72	4.21	0.023
Rape myth score (mean) (high = more myth belief)	450		9.89	9.59	10.20	1176		9.42	9.20	9.64	0.013
**Practices of hegemonic masculinity and gender relations**											
>20 sexual partners	238	451	52.8	47.9	57.6	304	1203	25.3	22.6	27.9	<0.0001
Any transactional sex	366	466	78.5	74.8	82.3	717	1220	58.8	55.4	62.1	<0.0001
Physical IPV: never	143	446	32.1	27.4	36.7	782	1160	67.4	64.7	70.1	<0.0001
once	71		15.9	12.3	19.5	142		12.2	10.4	14.1	
more than 1 time or type	232		52.0	47.0	57.0	236		20.3	18.0	22.7	
**Substance abuse and gang membership**											
Alcohol consumption: none	92	430	21.4	17.0	25.8	407	1161	35.1	32.1	38.1	<0.0001
moderate	186		43.3	38.3	48.2	514		44.3	41.5	47.0	
high	152		35.3	30.3	40.4	240		20.7	18.0	23.3	
Drug use in the past year	246	437	56.3	50.9	61.7	373	1180	31.6	28.4	34.8	<0.0001
Gang membership	97	437	22.2	18.0	26.4	82	1178	7.0	5.2	8.8	<0.0001
**Psychological measures**											
Life circumstances now less good than peers	119	424	28.1	23.8	32.3	192	1138	16.9	14.6	19.1	<0.0001
Machiavellian egocentricity & blame externalisation (mean)	418		33.61	32.71	34.51	1113		28.24	27.61	28.87	<0.0001
Empathy (mean)	412		0.45	0.41	0.50	1102		0.55	0.51	0.59	0.001

Men who raped rated as poorer than that of their peers their achievement in current life circumstances. They also scored much higher on the measure of blame externalisation and Machiavellian Egocentricity. They had lower levels of empathy.

In terms of substance use (table 2), men who raped consumed more alcohol and many more had used drugs (dagga). Nearly a quarter (22%) of had been in a gang which was much more common than among men who had not raped.

Table 3 presents the findings of analyses of questions on a range of different anti-social, criminal and violent practices. It shows that rape perpetrators' violence against women, is just one dimension of a broader spectrum of violent and anti-social practices. Men who had raped were much more likely to have bullied others at school, to have stolen on multiple occasions or handled stolen goods, a quarter (24.1%) had had possession of an illegal firearm, a third (31.3%) had another weapon, one in ten (9.5%) had also raped a man, and nearly a third (29%) has been arrested for another offence. All these practices were much more frequently reported by men who had raped than those who had not, except possessing a licensed gun.

**Table 3:** Distribution of engagement in other violent and anti-social behaviours by rape perpetration.

	Rape					Never raped					
	n	N	%	95% CI		n	N	%	95% CI		p value
School bullying score (mean)	454		12.29	11.90	12.68	1191		9.90	9.75	10.06	<0.0001
Stolen something or had stolen goods: Never	99	432	22.9	19.2	26.6	588	1167	50.4	47.2	53.6	<0.0001
1–2 occasions	82		19.0	15.1	22.9	294		25.2	22.5	27.9	
3 or more	251		58.1	53.2	63.0	285		24.4	21.7	27.2	
Rape of a man	44	462	9.5	6.7	12.4	6	1211	0.5	0.1	0.9	<0.0001
Arrested for another crime	100	345	29.0	23.9	34.0	245	1177	20.8	18.1	23.6	0.001
Ever in possession of an illegal gun	106	439	24.1	20.0	28.3	72	1176	6.1	4.5	7.8	<0.0001
Has a weapon other than a licensed firearm	136	435	31.3	26.7	35.8	189	1175	16.1	13.8	18.3	<0.0001
Has a licensed firearm	32	430	7.4	4.8	10.1	65	1167	5.6	4.1	7.0	0.219

Table 4 presents an age-adjusted logistic regression model of factors associated with having ever raped a woman (n = 466 men had raped). Having raped was associated with greater experience of adversity and trauma in childhood and likelihood of having been a victim of rape by a man. It was associated with greater maternal education, but much less equitable views on gender relations. Raping was associated with exaggerated performance of heterosexuality seen in terms of having had more partners, and ,much more inequitable practices of gender relations, notably with transactional sex and having been (more) physically violent towards a partner. Raping was also associated with drug use in the last year and with having been in a gang. Men who raped were more likely to have perceived that life had treated them more harshly than their peers, and scored higher on the dimensions of psychopathic personality, namely blame externalisation and Machiavellian egocentricity.

**Table 4:** Logistic regression models of a) factors associated with having ever raped, and b) factors associated with having raped in the past year.

	Model A				Model B			
	OR	95% CI		p value	OR	95% CI		p value
Childhood trauma scale	1.04	1.01	1.07	0.012	1.04	1.00	1.09	0.04
Raped by a man	2.18	1.35	3.52	0.002	1.97	1.02	3.82	0.044
Mother's education: none	1.00							
some schooling	1.96	1.26	3.04	0.003				
completed school or higher	4.24	2.29	7.85	<0.0001				
Gender Equitable Men scale	0.97	0.94	1.00	0.024				
>20 sexual partners	1.82	1.32	2.52	<0.0001				
Ever had transactional sex	1.53	1.04	2.23	0.029				
Physical IPV perpetration: never	1.00				1.00			
once	1.77	1.12	2.80	0.015	2.53	1.10	5.82	0.029
more than 1 time or type	2.82	1.98	3.99	<0.0001	3.36	1.75	6.48	<0.0001
Past year drug use	1.50	1.09	2.07	0.013				
Ever a gang member	1.88	1.19	2.96	0.007				
Life circumstances less good than peers	1.66	1.14	2.42	0.008				
Machiavellian egocentricity & blame externalisation	1.03	1.01	1.05	0.001	1.06	1.03	1.09	<0.0001

The model of rape perpetration in past year (78 men disclosed past year rapes) showed this to be associated with men's exposure to trauma in childhood, having been themselves raped by a man, greater blame externalisation and Machiavellian egocentricity and perpetration of physical violence towards a woman partner.

### Motivations for raping

**Figure 2:**
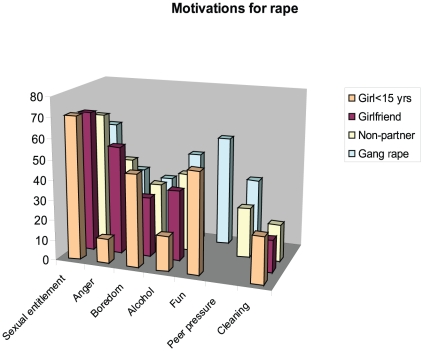
Graph of motivations for rape by victim type or circumstance.

Figure 2 shows the motivations for rape by type of rape. The most common motivations for all types of rape stemmed from ideas of sexual entitlement, a further measure of which is that 45% of men indicated that they had felt no guilt about their act of rape. Rapes of girls under 15 were largely a product of boredom and undertaken for ‘fun’. Gang rapes were similarly perpetrated from boredom and for ‘fun’ but the men also said they had often been drinking and many that they had been pressurised into participation, and for some the rape was an act of punishment of the victim, motivated by anger (c.f. Wood [58]). Women who were strangers or acquaintances, as well as girlfriends, were often raped as punishment, but also in contexts where men were bored, were pressurised by friends and had been drinking. Punishment was more common as a motive for rape of girlfriends. Only in a minority of cases did men say that their act of rape had been motivated by a perceived need to cleanse themselves of diseases though this was more common in rapes of young girls. Men who had raped young girls were asked if part of the explanation was that they ‘thought they would not tell’ and 49.3% indicated affirmatively.

## Discussion

This study of rape perpetration was conducted with a representative sample of men from the community, and it is notable for the large sample size. The prevalence of rape perpetration disclosed in the survey was very high, with more than one in four men disclosing having raped a woman and 15% of men had raped on more than one occasion. However, it was not very different from the finding of Abbey et al [12] in their small community-based study of American men and is well within the range of victimisation experiences reported by women across the globe [3]. The prevalence was comparable with that reported by South African men aged 15–26 who were participating in the Stepping Stones Trial (21%) [10] and was lower than the prevalence found in a representative random sample of men living in Gauteng Province in South Africa (37.4%) [59]. The past year rape perpetration prevalence in the latter study was 4.7%, which is the same as that reported here [59].

In research internationally, rape of or by an intimate partner is much more commonly reported than non-partner rape [3]. Our findings are notable in that men more often disclosed rape of a stranger or acquaintance than a partner. This finding is supported in the other datasets in South Africa [10], [59]. The prevalence by age group suggests that there has been no change in prevalence over the period of men's lives reported in the study. Indeed the data may well provide insights into rape over about 30 years as most men who had raped had done so for the first time as teenagers. A similar finding has been described from research in the United States [60], and is implicitly supported by the high prevalence of rape among young South African men [10]. This shows that primary prevention of rape perpetration needs to focus on young teenagers.

The analysis of factors associated with having raped modelled all types of rape. Unlike other authors, we did not model rape of a partner separately, because we did not distinguish rape of a non-partner from that of a partner because of the considerable overlap between rape victim categories. Where it has been done, little difference in associated factors was found [10].

The men who raped differed from those who had not in five important ways. First they had had a much greater exposure to abuse and adversity in childhood. This confirms the findings of Malamuth et al [13], [14] and emphasis placed by Knight and Sims-Knight [27]. Secondly, they were more likely to have a pattern of ideation that would enable them to justify rape. They held more gender inequitable views, adversarial and hostile ideas about women and were more likely to subscribe to rape myths that provide cognitive legitimacy for their acts. Again this has been described in the Confluence Model. Thirdly they had engaged in a pattern of behaviour with female sexual partners that has been identified as rooted in hegemony masculinity and indicates a need to display exaggerated heterosexual performance and to control women [47], [49], [61]. They had very large numbers of sexual partners, and had engaged in transactional sex, (a practice understood as part of a pattern of control of women), rather than stemming from a desire to emotionally engage with them [14], [49]. The use of physical violence against partners is also symptomatic of this, and we have shown that men who rape are much more likely to be physically violent towards women (c.f. Jewkes et al [10]). Fourthly men who raped saw themselves as victims. They felt that they had not got what they deserved from life, their peers had attained more, and perhaps that they possessed less than they should have given their mother's relatively high educational status. They externalised blame, whilst acknowledging that they deliberately tested boundaries. The association with dimensions of psychopathy confirm the importance placed on this by Knight and Sims-Knight [27]. Finally, they were more likely to have been, or currently be, engaged with delinquent or criminal peers, either as a member of a gang, using drugs, or participating in a range of other criminal and violent behaviour. An important finding also related to the relationship of rape and poverty as this was clearly complex, with the very poorest and socially disadvantaged men not being more likely to have done so.

The data on motivations for rape provides insights which are not available from the logistic regression models but in many respects triangulate their findings. They point to an essential disjunction between the criminal offence of rape, which is generally condemned in South Africa, and the acts of sexual coercion which were indistinguishable from the legal categories in intents and acts, but appear not to have been widely viewed as ‘rape’ or regarded as unacceptable and violent behaviour. In the research setting there are acts that have traditionally been considered ‘legitimate’ which involve coerced sex, although the legitimacy is increasingly contested. This is most notable in the traditional marriage practice of *ukutwala* (wife abduction), which is still to some extent practiced today (3 of the married men had married this way) and in recommended sexual cleansing after traditional circumcision, which to the extent that the sexual partner is supposed to be someone who the man does not care for, is rumoured to often take the form of rape (Sikweyiya: personal communication). The most commonly reported motivations stemmed from ideas of sexual entitlement and of rape motivated by anger and a desire to punish. This finding strongly supports the need to emphasise gender inequality and gender relations in understanding the act of rape and the patterns of ideation that provide space for it. Important here is a sense that in particular circumstances the sexual act was viewed as legitimate, at least in local or sub-cultural terms, a perception that would have been strengthened in the context of gang rape by group participation. Our finding that nearly one in five of the men interviewed had participated sexually or in another way in gang rape further shows that among men of a particular age, gang rape perpetration is widely viewed as something ‘boys do’. This helps to explain both the high prevalence of rape perpetration and the willingness of men to disclose it in a survey.

Alcohol was seen as a contextual factor, with having been drinking ascribed as one of the motivations for rape of other women. This has been described in North America [16], [23]. However men who drank more were not more likely to rape after adjusting for other risk factors. This rather supports the limited attention that some previous authors have given to alcohol [13].

The patterns of motivation for rape of girls under age 15 were somewhat different from that of older women. It is likely that the perpetrators were a mixed group. Some will have been young themselves, merely selecting victims who were younger than them (or finding themselves in situations in which the victim had been selected by their peers), and some are likely to have been older men who were sexually attracted towards children. The much lower likelihood of anger and alcohol being involved reflects both of these groups [62]. Cleaning of sexual diseases was more often a motivation for rape of young girls but the findings suggest that this is in most cases not seeking a virgin cure for HIV but rather a more general notion of sex as cleansing which is prevalent in the ethnomedicine of this area [63]. Only three men in this study who were HIV positive, had previously tested for HIV and taken their result, and who disclosed rape of a girl under 15 were motivated by cleansing. None of these had ideation supporting the virgin cleansing idea although two said they would deliberately spread of HIV if they knew they were infected [64].

Our findings have considerable resonance with those from North America that gave rise to the Confluence Model [14] , but they suggest that this model has an important weakness in its failure to identify the important role of patriarchy and the gendered value system which is embodied in cultural ideas of manhood that legitimate men's control over women as of critical importance in understanding rape perpetration. Our findings provide very considerable support to feminist analyses of rape [e.g. 29], [30], [65]. They suggest that changing the national gender order and inequitable constructions of masculinities *per se* is required for rape prevention.

Our findings also support the critical importance of exposure to childhood trauma and victimisation, in rape perpetration causation. There are three explanations for how this may act to impact on rape perpetration. The first is a neurophysiological explanation, in that trauma produces a cascade of physiological and neurohumoural responses that lead to enduring patterns of brain development and alterations in functioning that result in less impulse control and more self-destructive and abusive behaviours [66]. A psychoanalytic explanation is that impaired and disorganised attachment, particularly to mothers, due to parental absence, use of harsh parenting practices and emotional harshness in childhood impacts on boys' psychological development lowering their self-esteem and impacting on personality development, particularly the risk of borderline personality and narcissism, both of which are associated with lower empathy and a greater propensity for rape [31]. Previous researchers have found that empathy is protective against rape perpetration [16]. Although we found it lower among men who raped, it was not found to protect against raping after adjusting for other factors. A third explanation is that men who have been exposed to trauma may admire and seek the company of delinquent peers who engage in acts that explore their power and its boundaries which are criminal and involve interpersonal violence. This is shown in the association established in historical and ethnographic work between gang membership and raping [10], [67], [68]. Gangs are well recognised for providing an emotionally supportive peer context for acts of violence and inter-personal dominance, and ones associated with an aggressive, anti-social exaggerated masculinity [32], [67].

Discussing rape myth acceptance and sex role stereotyping in rape, many authors have suggested that this is exaggerated, and in particular they have commented that they have distributions in convicted rapists that are little different from other men [13], [54], [69], [70]. Our findings confirm them to be highly prevalent, but we argue that they should be understood as providing legitimation for rape that is part of the ideational context of patriarchy and enables rape to be cognitively justified. This is important for intervention even if it is not sufficient alone to explain specific acts of rape.

The main strength of this study is that it involved a large randomly selected sample of adult men from the general population, the questionnaire included item to capture all the main variables previously found to be associated with rape and the survey had a good response rate. The findings should be generalisable. Under-reporting of rape and other anti-social behaviours in the survey is a risk, but we hope that the confidential interview process with self-completion of the questionnaire will have minimised this. Indeed the high level of reporting of rape suggests some success. The response rate was good, but non-respondent bias remains a risk. The men interviewed were younger than men in the population over all and there was a high refusal rate in areas where the population was predominantly White (48.6%). It's important that the analysis showing weighted prevalence adjusted to the Provincial age-distribution estimates was very similar to the unweighted prevalence. We were not able to weight for race because we do not know the anticipated racial distribution of the study area. The White areas with a high refusal rate only constituted 3.6% of EAs and so are unlikely to greatly influence the prevalence estimates.

The study was cross-sectional and so it is impossible to be sure of the temporal sequence of many of the factors associated with raping. It is possible that attitudes towards women and gender relations were formed as a post-hoc cognitive justification of the act. However given that our formative research showed that many men did not think that they had ‘raped’ when disclosing acts of sexual coercion of women, this is unlikely to be a major consideration [50]. Childhood factors are likely to have preceded the first act of rape and definitionally preceded rape reported in the previous year. Psychoanalytic literature on personality disorders suggests that psychopathy is likely to develop from early childhood experiences and may be genetically influenced [31]. Fewer variables were associated with rape perpetration in the past year than in the lifetime, and those seems to be most easily explained by the fact that this act was relatively low prevalence (4.7%, n = 78) and so cell sizes were smaller.

### Conclusions

Our findings show rape perpetration to be highly prevalent in South Africa and they underline the importance of focusing on rape perpetration in strategies for rape prevention. Broad approaches to primary rape prevention are required. The two most important foci for rape prevention should be on men's childhoods, reducing exposure to trauma through strengthening parenting and on changing the ideals of men's gender socialisation, i.e. addressing sexual entitlement, gender hierarchy, legitimacy of punishment and use of violence to assert power over women. Furthermore they point to the critical importance of targeting interventions at teenagers, and engaging with the connections between youth anti-social and petty criminal behaviour and perpetration of very severe acts of violence. The law has an important contribution to make in asserting social values, but the very widespread practice of rape cannot be changed by criminal justice responses alone, especially as in all settings they seem to result in few convictions [71], [72].
